# Targeting integrin α5β1 in urological tumors: opportunities and challenges

**DOI:** 10.3389/fonc.2023.1165073

**Published:** 2023-07-06

**Authors:** Xuming Zhou, Hezhen Zhu, Cong Luo, Huan Xiao, Xiaofeng Zou, Junrong Zou, Guoxi Zhang

**Affiliations:** ^1^ The First Clinical College, Gannan Medical University, Ganzhou, China; ^2^ Department of Urology, The First Affiliated Hospital of Gannan Medical University, Ganzhou, China; ^3^ Institute of Urology, The First Affiliated Hospital of Gannan Medical University, Ganzhou, China; ^4^ Jiangxi Engineering Technology Research Center of Calculi Prevention, Ganzhou, China

**Keywords:** integrin α5β1, prostate cancer, bladder cancer, kidney cancer, inhibitors, drug resistance, target

## Abstract

Urological tumors, such as prostate cancer, renal cell carcinoma, and bladder cancer, have shown a significant rise in prevalence in recent years and account for a significant proportion of malignant tumors. It has been established that metastasis to distant organs caused by urological tumors is the main cause of death, although the mechanisms underlying metastasis have not been fully elucidated. The fibronectin receptor integrin α5β1 reportedly plays an important role in distant metastasis and is closely related to tumor development. It is widely thought to be an important cancer mediator by interacting with different ligands, mediating tumor adhesion, invasion, and migration, and leading to immune escape. In this paper, we expound on the relationship and regulatory mechanisms of integrin α5β1 in these three cancers. In addition, the clinical applications of integrin α5β1 in these cancers, especially against treatment resistance, are discussed. Last but not least, the possibility of integrin α5β1 as a potential target for treatment is examined, with new ideas for future research being proposed.

## Introduction

Cancer remains one of the major contributors to death, with an estimated 19.3 million new cases and nearly 10 million cancer case deaths worldwide in 2021. Among the most prevalent cancers, prostate cancer ranks first, bladder cancer fourth, and renal cell cancer sixth in incidence rates among men (1). The main treatment for these diseases is surgical resection, but the outcomes are often unsatisfactory, mainly due to high recurrence rates (50% for bladder cancer and 40% for renal cell carcinoma) (2–4). During the treatment of prostate cancer, 80% of patients are initially highly sensitive to androgen deprivation therapy, but almost all enter the castration-resistant stage of prostate cancer after treatment (5). Metastatic disease is reportedly the leading cause of death from urological tumors, with lymph nodes surrounding the primary tumor being the primary target of metastasis, followed by metastases to the liver, lungs, and bone (6). Metastasis affects a patient’s quality of life and has an extremely poor prognosis since it can directly result in death. Understanding the mechanisms of tumor metastasis is crucial for controlling disease progression and prolonging the survival of this patient population.

Integrins are a family of cell surface receptors composed of α and β subunits that form heterodimers through non-covalent bonds and are expressed in most cells, including endothelial cells, fibroblasts, pericytes, and tumor cells (7, 8). Integrins are important regulators *in vivo*, mediating cell binding to the extracellular matrix and producing signals associated with various diseases, such as autoimmune responses, tissue and organ development, cardiovascular disease, and cancer (9). In humans, integrins consist of 18 α-subunits and eight β-subunits, forming 24 heterodimers. The α-subunits are mainly associated with receptor recognition and contribute to binding integrin receptors with cation-dependent fits. The β-subunits are associated with cell-to-mesenchyme and cell-to-cell signaling and are involved in cytoskeletal protein interactions and intracellular signaling (8, 10, 11). The α and β subunits both have a long extracellular structural domain and a short cytoplasmic tail; the cytoplasmic tail is connected to the actin cytoskeleton and intracellular signaling pathways such as SRC protein family kinases, focal adhesion kinase (FAK), Rho-GTPase family, mitogen-activated protein kinase (MAPK), protein kinase B (AKT), and integrin-linked protein kinase( (12). Integrins are important regulatory factors in the differentiation, metastasis, angiogenesis, and immune escape of tumor cells *in vivo* and tumor radiotherapy resistance.

## Structure, function, and ligands of integrin α5β1

Integrin α5 is one of the 18 subunits of integrin α, which usually forms a heterodimer with integrin β1. Integrin α5β1 belongs to a family of 24 heterodimers of integrins, consisting of two subunits, α5 and β1 (13, 14). The human integrin α5 gene encodes the α5 subunit, localized at 12q11. The α5 subunit has an extracellular leg structural domain and a β-helix structural domain responsible for recognizing the arginine–glycine–aspartate structural domain (RGD structural domain) on fibronectin and fibrinogen (15). The β1 subunit is located at the chromosomal region 10p11.2 and consists of a follower protein–signin–integrin (PSI) structural domain, a heterodimeric domain, a β1 structural domain, and four epidermal growth factor-like structural domains (16). It is now understood that integrin α5β1 is dependent on the MIDAS (with metal ion-dependent adhesion sites) structure and divalent cations to interact with extracellular ligands, and calcium ions are important cations for integrin α5β1 ligand binding (17).

The integrin α5 subunit usually binds to the β1 subunit to form a heterodimeric integral membrane protein, the only known α5 integrin (18). After binding to the integrin α5β1 cytoplasmic tail and associated ligands, it binds to the cytoskeleton and drives cytoskeletal reorganization through an outside-in signaling pathway. Integrin α5β1-regulated intracellular signaling activates the extracellular compartment and assists in assembling the extracellular matrix, i.e., an inside-out signaling pathway (19). This bidirectional signaling pathway involves biological behaviors such as cell adhesion, migration, and survival (20). In addition, integrin α5β1 can act as a pro-angiogenic factor that is involved in tumor angiogenesis by interacting with vascular endothelial growth factor receptor and angiopoietin and has received a great deal of attention for its importance in tumorigenesis, metastasis, and drug resistance (21).

## Integrin α5β1-related ligands

Current evidence suggests that integrin α5β1 plays an important role in tumor metastasis, and cell membrane ligands initiate cancer cell invasion by regulating α5β1 activation. Given that integrin α5β1 can recognize and adhere to extracellular ligands with RGD structural domains, most of its ligands have RGD structures. Fibronectin, fibrinogen, and fibrin-1 are the main ligands of integrin α5β1 and, in addition to mediating cell proliferation, migration, and differentiation effects, promote fibronectin polymerization and assembly into a matrix and have a potential role in proliferation and invasion in urological tumors (9, 22–25). Interestingly, endothelial cells secrete soluble vascular endothelial growth factor receptor-1, a stroma-associated protein that interacts with integrin α5β1 and plays an important role in angiogenesis in cancer (26). Other related ligands containing RGD structures include Adgre5, UPAR, and TRAP, which interact with integrin α5β1 to induce intracellular signaling, migration, and angiogenesis in tumor cells (27–29). Related ligands for integrin α5β1 also include porcine hemagglutinating encephalomyelitis virus, 25-hydroxycholesterol, tubulointerstitial nephritis antigen-like 1, pregnancy-specific glycoprotein 1, and neuropilin, among others (30–34). These studies overlap in their assertion that these ligands play important roles in cell adhesion, invasion, proliferation, and angiogenesis during binding to integrin α5β1 (**Table 1**).

**Table 1:** Ligands and roles of integrin α5β1.

Ligand	Functions	Reference
Fibronectin	Mediates cell proliferation, migration, and differentiation	(21)
Fibrinogen	Mediates cell proliferation, migration, and differentiation	(22)
Fibrillin-1	Mediates cell proliferation, migration, and differentiation	(23)
VEGFR-1	Regulation of angiogenesis, metastasis, and drug resistance	(24)
Adgre5	Mediates cell adhesion, migration, and angiogenesis	(26)
UPAR	Induces intracellular signaling, migration, and angiogenesis	(28)
TRAP	Induces intracellular signaling, migration, and angiogenesis	(27)
PHEV	Regulation of actin cytoskeleton rearrangement	(30)
25-Hydroxycholesterol	Mediates cell signaling and adhesion	(31)
TinaGL1	Inhibition of integrin/FAK and EGFR signaling pathways	(32)
PSG1	Regulation of extrachorionic trophoblast migration	(33)
Neuropilin	Induction of cell migration	(29)

## Role of integrin α5β1 in common urological tumors

The aberrant expression of integrins has been associated with the development of urologic tumors and their poor prognosis. Integrin–extracellular matrix interactions play a key role in cell adhesion. As a transmembrane protein, integrin α5β1 possesses extracellular, transmembrane, and cytoplasmic structural domains, where the extracellular and transmembrane structural domains are responsible for binding to extracellular matrix proteins or other extracellular ligands and participate in subsequent signaling pathway functions, while the cytoplasmic structural domain can interact with cytoskeleton-associated proteins and affect cell migration, invasion, and proliferation (9, 31, 35–41). The functional role of integrin α5β1 in common urological tumors will be briefly described below.

### Prostate cancer

It has been established that, in prostate cancer, there is a correlation between altered integrin expression and abnormal extracellular matrix secretion, progression, and invasion (42, 43). Several studies have reported the dysregulation of both integrin α and β subunits during prostate cancer progression (44, 45). Fibronectin polymerization is an important regulator of extracellular matrix stability (25, 46). A study revealed that the blockade of integrin α5β1 with the proline–histidine–serine–arginine–asparagine (PHSCN) peptide significantly prevented cell metastasis in preclinical prostate cancer models and in phase I clinical trials conducted in parallel (47). In an *in vitro* study, it was shown that integrin α5 plays an important role in the adhesion and spreading of PC-3 prostate cancer cells interacting with fibronectin, and blocking integrin α5 caused a decrease in the number of adherent cells in the early stages of adhesion, diminished cell extension kinetics, and cell morphology changes. Besides these, cytoskeletal protein reorganization was diminished. Moreover, the blockade of integrins using the fibronectin-related peptide GRGDSP (Gly-Arg-Gly-Asp-Ser-Pro) completely inhibited the growth and cell morphological alterations of prostate cancer PC-3 cells, confirming that integrins interact with the FNIII10 structural domain and play a key role in these processes (48, 49). The downregulation of the β1 integrin subunit has been shown to significantly reduce the expression of the relevant α-subunit in prostate cancer cell lines (50). Moreover, in the absence of type 1 insulin-like growth factor receptor, integrin α5β1 was transported to the proteasome and lysosome for degradation rather than transferred to the intranucleosome for recycling, and type 1 insulin-like growth factor receptor signaling controlled the stability of integrin α5 through the proteasome pathway, thereby regulating the stability of prostate cancer pro-survival signaling (50, 51). The inhibition of the α5 subunit *in vivo* has been shown in other studies to significantly inhibit tumor growth (52, 53). In conclusion, integrin α5 plays an important role in prostate cancer progression, and it can be inferred that integrin α5-related inhibitors may contribute to blocking tumor progression.

### Bladder cancer

Bladder cancer is one of the most common urinary tract cancers, ranking ninth in the global incidence of cancer (54). Integrin α5β1 has also been correlated with the development and progression of bladder cancer. Zhou et al. (55) found that the interaction between integrin α5 and fibronectin could be affected by the sialidase NEU1, and NEU1 overexpression decreased the levels of fibronectin and integrin α5 in the plasma membrane, increased the degradation of fibronectin by lysosomes, and inhibited the downstream AKT pathway. These processes suppressed cancer cell proliferation, induced apoptosis, and inhibited tumor formation *in vitro* and *in vivo*, thereby inhibiting bladder cancer progression. Laidler et al. (56) found that the expression of integrin subunits α5 and β1 was significantly higher in malignant bladder cancer cells Hu456 and T24 than in non-malignant uroepithelial cell HCV29, suggesting that changes in integrin α5β1 expression may also contribute to bladder metastasis cell carcinoma progression, invasion, and metastasis. It has been shown that the adhesion of BCG to bladder cancer tumor cells is mediated by fibronectin (57). The direct antitumor effect of BCG in the treatment of bladder cancer is initiated by binding to the fibronectin receptor integrin α5 (58). The correlation between high integrin expression and bladder metastatic cell carcinoma was consistent with the antitumor properties of BCG (59). Kato et al. (60) found that BCG exhibited antiproliferative effects only in integrin α5-positive T24 and HT1376 cells but not in RT4 cells lacking integrin α5 on their surface, demonstrating that the antitumor effect of BCG on bladder cancer is at least partially dependent on the biological function of integrin α5.

### Kidney cancer

Kidney cancer is reportedly the 12th most common cancer globally, with six cases per 100,000 men and three cases per 100,000 women diagnosed with kidney cancer yearly. The incidence is estimated to increase by 2.4% annually (61). Integrin α5 is expressed at significantly higher levels in renal clear cell carcinoma than in normal tissue and plays an important role in renal cancer progression (62). Hase et al. (63) showed that the LOX-like protein (LOXL2) promotes tumor progression by regulating integrin α5 levels through protease and proteasome-dependent pathways. Haber et al. (64) found that increasing integrin α5 levels and downstream signaling through AKT could help tumor cells adhere to extracellular matrix compounds and promote bone metastasis in renal cell carcinoma. It has also been shown that, by inhibiting integrin-linked kinase, the quinazoline-derived drug DZ-50 could significantly inhibit the metastasis of renal cancer by blocking the phosphorylation of AKT and FAK and subsequent cell survival, disrupting the adhesion of integrin α5, and killing tumor cells by exposure to extracellular matrix-associated tumor suppressors (65).

## Mechanism of integrin α5β1-mediated tumor proliferation, metastasis, and drug resistance

**Figure 1:**
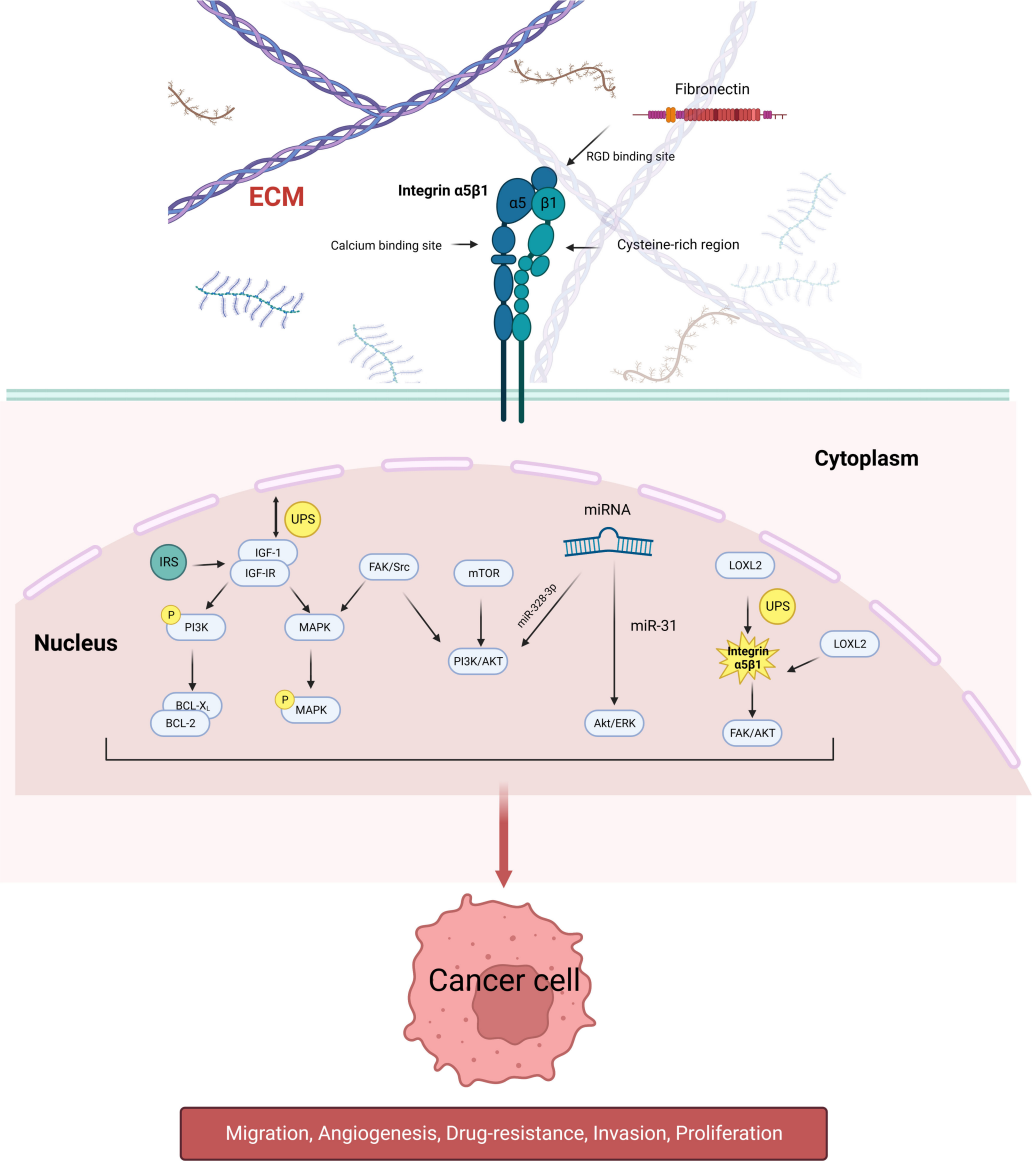
Schematic diagram of the signaling pathways involved in integrin α5β1 that mediate tumor cell proliferation, migration, angiogenesis, and drug resistance. Integrin α5β1 contributes to cancer progression by activating the PI3K/AKT, MAPK, and ERK signaling pathways. LOXL2 protein regulates the stability of integrin α5β1, which is also the downstream target of many miRNAs. Integrin α5β1 is also involved in mTOR inhibitor resistance through the FAK/Src axis.

Integrin α5β1 does not directly regulate tumor proliferation, metastasis, and drug resistance but by a combination of extracellular ligands and intracellular signaling pathways. Some mechanistic studies on integrin α5β1-mediated urological tumors in related aspects are briefly described below (**Figure 1**).

### PI3 and MAPK signaling pathways

Insulin-like growth factor 1 (IGF-1) is a single-chain polypeptide that mediates endocrine, autocrine, and paracrine growth, thus acting as a potent growth factor. IGF-1 acts on cells by binding to its receptor, IGF-IR, a transmembrane protein with tyrosine kinase activity (66). The insulin receptor substrate protein (IRS) is a specific docking protein for IGF-IR and insulin receptor (IR) (67). It has been established that IRS1 and IRS2 do not contain intrinsic kinase activity but function by recruiting proteins to surface receptors and assembling them into signaling complexes. Signals from IRS proteins lead to the activation of pathways, including phosphatidylinositol-3 kinase (PI3K) and mitotic-activated protein kinase (MAPK) (68). Interestingly, these two pathways can be activated by integrins (69, 70). The link between integrins and IRS1 has been suggested as a possible mechanism for the synergistic effect of growth factors and extracellular matrix receptors (71). IGF-1 signaling is reportedly regulated by a negative feedback mechanism through ubiquitin/proteasome-mediated OFIRS2 degradation, which regulates the magnitude and duration of the response to insulin or IGF-1 (72). The integrin β1 subunit regulates IGF-IR expression and is essential for IGF-1-mediated androgen receptor (AR) activity (73). The downregulation of IGF-IR leads to a significant reduction in integrin α5 and β1 subunits; the inhibition of α5 integrin *in vivo* can significantly reduce tumor growth (74). This phenomenon may be attributed to the fact that IGF-IR regulates the stability of integrin α5β1 via the proteasome pathway, which modulates pro-proliferative signaling in prostate cancer cells (51).

### PI3K and FAK/AKT signaling pathways

Adhesion to fibronectin and its fragments, migration, and invasion of prostate cancer cells via integrin α5 is considered one of the mechanisms by which bone marrow localization is regulated by bone-derived mesenchymal stromal cells (49, 75, 76). Integrin-mediated cell adhesion to extracellular matrix components is an important regulator of tumor cell survival (77). Reducing the expression of the BCL-2 family and inducing apoptosis in PC-3 cells by knocking down integrin α5, the combined inhibition of the PI3K signaling pathway and integrin α5 enhanced apoptosis in these PTEN mutant cells. Furthermore, when BCL-2/BCL-XL was inhibited, the transcription and expression of integrin α5 were upregulated (78), which may be attributed to the fact that the synergistic inhibition of PI3K and integrin α5 leads to a reduced expression of the BCL-2 family downstream of integrin α5, mediating apoptosis in prostate cancer cells.

Cytoskeletal organization and adhesion formation are essential for cell motility and structural support (19). Integrins link the ECM to the intracellular cytoskeleton and adhesion foci, thereby controlling multiple signal transduction pathways, including cell proliferation and survival (79). Wang et al. showed that the decreased expression of integrin α5 contributed to the inhibition of lung colonization in bladder cancer, and depletion of eIF3b inhibited integrin α5 expression, suggesting that integrin is an important target leading to actin skeleton and adhesion disruption. The decrease in integrin yielded a similar effect on FAK and AKT phosphorylation (80). Similarly, the upregulation of integrin β1 induced FAK phosphorylation, leading to prostate cancer progression (81).

### LOXL2

LOX-like protein 2 (LOXL2) is a member of the lysyl oxidase family, consisting of Lox and four Lox-like proteins, with intracellular and extracellular functions. The secreted LOXL2 regulates integrin levels to promote tumor progression in renal clear cell carcinoma (ccRCC) by regulating lysyl oxidase LOXL2 status and its correlation with tumor staging (82). It was shown that LOXL2 and integrin α5β1 were significantly higher in ccRCC tissues than in normal kidney tissues, and LOXL2 was involved in the stabilization of integrin α5β1 protein in ccRCC cells. The RNAi-mediated knockdown of LOXL2 significantly inhibited stress fiber and adherent plaque formation in ccRCC cells. In addition, LOXL2 siRNA knockdown significantly inhibited cell growth, migration, and invasion. Mechanistically, it regulated the degradation of integrin α5β1 through proteasomal and proteasome-dependent systems (63). Collectively, these findings suggest that LOXL2 is a potent regulator of integrin α5β1 protein levels and has a pro-tumor effect in ccRCC.

### MicroRNA

MicroRNAs are endogenous non-coding RNAs found in eukaryotes that have regulatory functions. miRNAs consist of 21–25 nucleotides, are extensively involved in the pathogenesis of human cancers, and can function as oncogenes and tumor suppressors (83). Integrin α5 has been shown in previous studies to be a downstream target of many miRNAs, such as miR-26a, and miR-148b (84, 85). In bladder cancer, it was shown that integrin α5 and miR-328-3p expression were negatively correlated, and targeting integrin α5 inhibited bladder carcinogenesis by miR-328-3p could prevent bladder carcinogenesis and progression by targeting integrin α5 and inhibiting the downstream PI3K/AKT signaling pathway (86). Xu et al. reported that miR-31 expression inhibited bladder carcinogenesis by downregulating integrin α5 as well as downstream cascade signaling to exert tumor-suppressive effects by overexpressing miR-31, leading to the inhibition of Akt and ERK phosphorylation, possibly secondary to the downregulation of integrin α5. When integrin α5 expression was restored, the Akt and ERK signaling pathways were re-activated (87). Therefore, inhibition of bladder cancer by targeting integrin α5 and downregulating the Akt/ERK signaling pathway activity represents potential approaches.

### Drug resistance

During clinical practice, drug resistance is commonly observed in the treatment of tumors, and studies have shown that integrin α5β1 is involved in this phenomenon. Juengel et al. found that integrin α5 was involved in the development of renal clear cell carcinoma resistant to mTOR inhibitors, characterized by quantitative changes in integrin α5 expression during drug resistance and coupled with altered molecular function of integrins, forcing renal clear cell carcinoma to shift from adhesion to migration (88). Similarly, in studies of resistance to mTOR inhibitors in prostate cancer, β1 integrins significantly triggered the migration of tumor cells, mediating the activation of the AKT signaling pathway and triggering cancer cell metastasis during the upregulation of β1 integrin expression (89). In studies investigating bladder cancer resistance to gemcitabine and cisplatin, integrin β1 expression was upregulated in both resistant cell lines, and when integrin β1 was inhibited, its adhesion and chemotaxis were reduced in both resistant cell lines (90). Wu et al. found that HHT exhibited a stronger inhibitory activity than cisplatin, carboplatin, and doxorubicin in acting on bladder cancer; integrin α5β1 played a role in the resistance of bladder cancer to HHT treatment by extinguishing the integrin 5β1-FAK/Src axis. This resulted in the downregulation of the MAPK/ERK and PI3/Akt signaling pathways, decreased cell–ECM interactions and cell migration, and ultimately inhibited tumor progression and potential tumor resistance following treatment (91). Therefore, the study of integrin α5β1 in tumor resistance may help to treat disease progression due to drug resistance and prolong the survival of these patients.

### Integrin α5β1 as a specific therapeutic target

Integrin α5β1 represents a promising therapeutic target for tumor angiogenesis and tumor cell expression. Although most studies involving α5β1 inhibitors are in the preclinical or pre-phase III clinical trial stages, many specific inhibitors have been successfully developed. These inhibitors hold huge potential for use against specific subgroups of aggressive tumors at this stage. Given that integrin α5β1 can enhance angiogenesis, the main function of these related inhibitors is anti-angiogenesis, with the development of specific antibodies and small peptides that target this pathway (**Table 2**).

**Table 2:** Integrin α5β1-related inhibitors and functions.

Inhibitor	Functions	Reference
ATN-161	Inhibits tumor growth, metastasis, and anti-angiogenesis	(91)
SJ749	Suppresses cell proliferation, anti-angiogenesis	(101)
JSM6427	Suppresses cell proliferation	(104)
ILA1	Suppresses cell adhesion and invasion, anti-angiogenesis	(108)
Volociximab	Anti-angiogenesis	(95)
MINT1526A	Anti-angiogenesis	(109)
BsAbα5β1/αv	Suppresses cell adhesion, migration, and clone survival	(100)

#### Atn-161

ATN-161 (Ac-Pro-Her-Ser-Cys-Asn-NH2) is a small pentapeptide-containing cysteine that mainly binds to the arginine–glycine–aspartate (RGD) structural domain and acts as an inhibitor of integrin α5 (92). After the ATN-161 (PHSCN) peptide terminal was acetylated and amidated, its stability and biological activity were increased by 30 folds (93). ATN-161 reportedly originates from a synergistic position of fibronectin and interacts with integrin α5β1. It has been shown that this peptide preferentially binds to activated integrin α5β1, blocking prostate cancer invasion *in vitro* and inhibiting prostate cancer growth, metastasis, and tumor recurrence. In phase I clinical trials, systemic PHSCN peptide monotherapy demonstrated good tolerability, with metastatic disease progression delayed by 4–14 months in one-third of the treated patients (94). In another study on this drug, the integrin inhibitor AT-161 combined with 5-fluorouracil was injected into a mouse colon cancer liver metastasis model, and the combined application of ATN-161 and 5-fluorouracil significantly reduced tumor load as well as the extent of liver metastasis (*P* < 0.02), with significantly fewer hepatic tumor microvessels in the ATN-161 and ATN-161, 5-fluorouracil groups than in the control and 5-fluorouracil groups (*P* < 0.05). The combination of ATN-161 and 5-fluorouracil was more effective than either treatment alone, with a significantly increased apoptosis rate of tumor cells, inhibited proliferation of tumor cells (*P* < 0.03), and improved overall survival rate (*P* < 0.03) (95). Overall, the integrin α5 inhibitor ATN-161 plays an important role in anti-angiogenesis and is expected to become an important therapeutic agent in inhibiting tumor progression and anti-angiogenic treatment regimens.

#### Volociximab

Volociximab is a chimeric human α5β1 antibody that consists of a variable region of mouse antibodies, including a complementarity-determining region against the α5β1 integrin, combined with a constant region of human IgG4 heavy chain and kappa light chain. In preclinical models, volociximab has shown the ability to prevent neovascularization by inhibiting fibronectin binding, has similar affinity and activity to integrin α5, and has been shown to be safe, effective, and tolerable (96). Moreover, it was well tolerated in phase Ib in patients with non-small cell lung cancer, phase II in patients with epithelial ovarian or primary peritoneal cancer, and phase II trials in metastatic renal cell carcinoma (97–100).

#### BsAbα5β1/αv

Joshi et al. prepared and analyzed a bispecific antibody (BsAbα5β1/αv) in prostate cancer that targets the degradation of both α5 and αv integrins. The results showed that this antibody was superior to monoclonal antibodies in eliminating prostate cancer cell adhesion, migration, and clonal survival (101).

#### SJ749

SJ749 is a non-peptide inhibitor of integrin α5 that inhibits angiogenesis by affecting endothelial cell adhesion and migration (102). It has been shown that SJ749 inhibited the adhesion of both cell types to fibronectin in a dose-dependent manner and inhibited the proliferation of A172 cells in its effect on two human astrocytoma cell lines, A172 and U87 (103). It was also reported that SJ749 inhibition of integrin α5 reduced the chemotherapy-induced premature senescence in human glioblastoma and promoted apoptosis in a functional P53 background (U87MG cells) (104).

#### JSM6427

JSM6427 is also a potent, highly specific inhibitor of integrin α5 (105). This drug was developed as an anti-angiogenic agent for treating age-related macular degeneration (106). It has also been shown to inhibit the attachment, migration, and proliferation of human retinal pigment epithelium (RPE) cells with fibronectin. This inhibition was followed by a reorganization of the RPE cytoskeleton with distinct features similar to the quiescent state of the cells (107). Another study showed that the integrin α5 inhibitor JSM6427 inhibited the growth of gliomas and reduced the density of microglia at the tumor margins. After injecting glioma cells into experimental mice for 21 days and then treating them with JSM6427 for 14 days, the tumor volume was significantly reduced compared to the control group (108).

#### Other

Many blocking antibodies have been developed against the interaction between integrin α5 and fibronectin—for example, IIA1, a functionally blocking mouse antibody to integrin α5, can inhibit angiogenesis, cell adhesion, invasion, and tumor cell survival *in vitro* (109). MINT1526A, a functionally blocking monoclonal antibody to integrin α5, has been used as an anti-angiogenic treatment. When combined with integrin α5 and vascular endothelial growth factor inhibition, MINT1526A was well tolerated in phase I clinical trials and has demonstrated efficacy (110) (**Table 2**).

## Challenges in the clinical application of integrin α5β1

Research on integrin α5β1 inhibitors in recent years has helped to demonstrate the specificity of integrins and the ability of these inhibitors to control multiple oncogenic pathways. The development of integrin inhibitor compounds is essential for developing new and more effective therapeutic options for treating urologic tumors. Most integrin α5β1 inhibitors are currently in phase 1 and phase 2 clinical studies, and it is essential to initiate phase 3 clinical trials as soon as possible (111). There are several α5β1 imaging probes for the molecular imaging of tumors, which provide new avenues for subsequent cancer diagnosis (112–116).

## Conclusion

This review describes the structure, function, and association of integrin α5β1 with prostate cancer, renal cell carcinoma, and bladder cancer as well as the clinical applications and research in these diseases. Integrin α5β1 is closely associated with cancer development, progression, and prognosis. Dysregulation of integrin α5β1 is strongly related to the development of urological tumors and may serve as an important indicator for evaluating invasion and migration. Additionally, the upregulation of integrin α5β1 can promote the development of drug resistance in cancer cells, which prompted the exploration of novel strategies for overcoming drug resistance in chemotherapy. The overexpression of integrin α5β1 has been documented in various tumors and reportedly contributes to tumor progression, making it a potential target for tumor imaging and an independent indicator of poor prognosis. Besides this, it has been extensively explored as a tumor suppressor in preclinical or clinical studies. Nonetheless, integrin α5β1 has not yet been established as a definitive molecular biomarker that can be applied to specific diseases, representing an area for future research. Therefore, research on integrin α5β1 can help to improve the diagnosis and treatment of this patient population.

## Author contributions

GuZ conceived the manuscript. XuZ searched publications and drafted the manuscript. HZ, HX, and CL edited the tables and figures. XiZ and JZ reviewed the manuscript and polished the grammar. All authors contributed to the article and approved the submitted version.
